# Transactions of the Pathological Society of Philadelphia

**Published:** 1840-03-14

**Authors:** 


### MEDICAL EXAMINER.

DEVOTED TO MEDICINE, SURGERY, AND THE COLLATERAL SCIENCES.

No. 11.] PHILADELPHIA, SATURDAY, MARCH 14, 1840. [Vol. III.

### TRANSACTIONS OF THE PATHOLOGICAL SOCIETY OF PHILADELPHIA.

*March 7th*, 1840.

The President, Dr. Gerhard, in the Chair.

*Endocarditis, connected with Articular Rheumatism—Rasping systolic sound of the Heart over the mitral valve—General Dropsy—Death—Autopsy—Remarks.* By C. W. Pennock, M. D.

E. Williams, a negress, aged 17, entered the wards of the Philadelphia Hospital, June 18th, 1839. With the exception of measles in the winter of 1838, followed by conjunctivitis, she had enjoyed perfect health until May, 1839, when she was attacked with articular rheumatism, which was first manifested in the shoulders, subsequently affecting all the articulations with the exception of the jaw. The pain, redness, and swelling of the joints were very great, and soon after the commencement of the disease, acute, lancinating pain "darting through her," was felt in the region of the heart, accompanied by an irritated, corded pulse, palpitations and dyspnœa.

Venesection, cupping, and the exhibition of colchicum, formed the basis of her treatment, and at the termination of a month, being much relieved, she requested her discharge.

She was obliged, however, to return in a few days; the dyspnœa, palpitation, and pain of the left chest much increased, accompanied by œdema of the feet and ankles. The œdema increased rapidly until anasarca became general, which was followed, in the course of the summer, by ascites. The latter complication was not accompanied by any evidence of peritoneal inflammation, but there were physical signs of enlargement of the liver.

The dropsical effusion was repeatedly removed by diuretics and purgatives, and although the diseased cardiac symptoms had been mitigated, they recurred with increased violence in the month of October. In the early part of November, symptoms of pleurisy, complicated the original affections, and soon after, the following note was taken.

*November 18th*, 1839. Present state.—Countenance distressed and anxious; dyspnœa very great, obliging the patient to be constantly in an upright posture; intelligence normal; skin warm, not perspirable; cheeks infiltrated; lips tumid; slight infiltration of the cellular tissue of the body; legs greatly swollen, pit upon pressure. *Abdomen* much enlarged, measuring across the umbilicus, forty-five inches in circumference; not painful upon pressure, except some tenderness of the epigastric region; percussion is flat in the lower two thirds of the abdomen, accompanied by evident fluctuation upon palpation; right hypochondriac region more developed than the left; liver, by percussion, extends an inch below the margins of the ribs. The appetite is good; thirst inconsiderable; tongue rose coloured, covered with aphthous elevations; follicles enlarged; fauces very red. Secretion of urine scanty, very turbid, loaded with a lateritous sediment, which is rendered soluble by heat, whilst nitric acid throws down a dense reddish brown precipitate. Three watery alvine dejections daily; the catamenia have been suspended since the first attack of rheumatism.

*Chest.*—Marked prominence of the lower half of the sternum, and of the region of the heart. The præcordial region is dull on percussion along the lower margin of the second rib, from the right side of the sternum towards the left, five inches; beneath the third rib, from the same point laterally to the left, six inches; and on the line of the fourth ribs, the dulness extends from the junction of the cartilage with the rib of the right side, towards the left, one inch beyond the left nipple, being seven and a half inches. Percussion of the præcordial region yields a flat sound in a trapezoidal space, of two by three and a half inches, extending downwards from the third cartilage the length of the sternum; and, laterally, from the right margin of that bone, to within a short distance of the left nipple. The impulse of the heart is very forcible at the apex. Only one sound, a prolonged systolic, (the first sound,) is heard at, below, and to the left of the left nipple, at which points it is strongly rasping; ausculting on the line of the fourth rib, from the left towards the right, the systolic sound becomes shorter, and clearer, the rasping less marked, and, near the left margin of the sternum, a faint second sound is distinguished, the rasping sound being heard with more intensity over these portions of the chest coinciding with the situation of the left ventricle of the heart, than it is on the cartilages of the ribs covering the right ventricle. At the aortic valves (middle of the sternum opposite the cartilages of the third ribs,) first sound, rough; the second very indistinct, but accompanied by a faint bellows murmur. Over the pulmonary valves, and above the second rib of the left side, the second sound is heard more plainly than elsewhere. Pulse at the wrist, and pulsation of the heart, coincide; pulse of small volume, sharp, corded, regular, one hundred and eight per minute. Slight movement in the subclavian veins from regurgitation from the right auricle.

The anterior parieties of the chest, with the exception of the præcordial space mentioned, is resonant on the left side, dull on the right. Respiration, which is generally feeble on the right side, is slightly rude beneath the clavicle; the respiration of the left chest is expansive immediately beneath the clavicle, but is masked elsewhere in front, by the violent impulse of the heart. Posteriorly, the left chest is most developed; the percussion is flat in the lower half of both sides, in the upper portion of the right it is obscure, and in the corresponding portion of the left chest, clearly resonant. Respiration, posteriorly, is vesicular in the upper part of the left lung; over the right, generally, feebly vesicular, except in the middle portion, and over the root of the lung, where it is tubal, with increased resonance of the voice. Along the right margin of the spine, the roughened sound of heart very perceptible, more so than along the left.

Occasional paroxysms of coughing; expectoration scanty; fluid aerated, not viscid. Neuralgic tenderness of the spine.

(The general treatment consisted in the frequent, though moderate, abstractions of blood by the lancet, or by cups upon the chest; the exhibition of digitalis, squills, and calomel; foot baths at night; bandaging anasarcous extremities. Diet farinaceous.)

The blood drawn was usually sizy, and the crassamentum was double the serum in quantity. The pulse became gradually less corded and frequent; on the 22d it was eighty-four per minute, corresponding with the number of the pulsations of the heart, and somewhat undulatory. The digitalis was then stopped, after a recent exhibition of thirty-six grains within the preceding ten days. The cardiac sounds were changed in character; the second sound, which had been scarcely audible, was, when the patient was tranquil, plainly heard over the aortic valves, and was occasionally distinguished, though feebly, over the mitral valve, although the rough bellows or rasping sound predominated. The second sound, however, became indistinct over the left side of the heart when the patient used any unusual exertion, or when mental agitation existed.

*Nov.* 23.—Pulse at the wrist, and pulsations of the heart, when first examined, beat at fifty-four per minute, and the two sounds of the heart distinguished over the aortic and mitral valves. Slight excitement produced by answering questions, increased the action of the heart, causing two systolic movements to one pulsation at the wrist; each systole is accompanied by moderate impulse. Accompanying each of these cardiac pulsations is a rasping sound, most marked between the third and fourth ribs of the left chest, three inches from the sternum, and also at, and to the left of the left nipple.

Over the aortic valves, the double systolic sounds are also heard; no second sound accompanies the first contraction of the ventricles, but a faint second sound is heard over these valves at the second systolic movement. Along the right margin of the sternum, and over the cartilages of the fourth and fifth left ribs, although the double movement is felt and heard, yet the roughened sounds do not exist, and become more feeble as we recede from the left portion of the heart.

Pulse at the wrist is of very small volume, thread like, easily obliterated. Countenance more anxious. Respiration costal, forty-eight per minute. Abdomen distended, but more flaccid; œdema of cellular tissue less; alvine and urinary secretions not increased. No vertigo, no ringing in the ears; sight and intelligence natural.

℞.—Camphor julep, eth. sulph. compos. and wine p. r. n. Rich animal broths.

The double stroke of the heart, with feeble arterial pulsations in the arm, generally forty-eight per minute, (the cardiac being ninety-six,) continued six days, attended by the peculiarities mentioned; and although neither the alvine or urinary discharges had increased, yet the dropsical fluids evidenty diminished.

On the 29th of November, the double stroke of the heart had disappeared; both sounds were clearly distinguished at the aortic valve; both sounds roughened, (saw sound.) No rasping sound either at the mitral valve, or at the apex. Pulse one hundred and twelve, of good volume and force. The ascites increasing, the patient was again put upon the use of digitalis and squill, of which gr. iij of the first, and gr. vj of the last, were exhibited daily.

On the 4th of December, patient had a chill followed by fever, acute pain in the right chest and in the præcordial region extending to the back, and increased dyspnœa. On the 6th percussion of the *right chest* was generally obscure; in the upper third posteriorly, nearly flat; respiration feeble anteriorly; posteriorly, in the upper third, it was bronchial, with fine crepitus; feeble with crepitus in middle third, scarcely heard below; the resonance of voice was very strong in the upper portion; in the lower half, it was hægophonic. *Left chest*, percussion in upper portion, exclusive of the præcordial region, was resonant, flat in the lower half; the respiration expansive, but roughened in the posterior upper third; below that, extremely faint, and masked by crepitus; resonance of voice increased. Recurrence of the rasping sound over the left ventricle during the systole of the heart, whilst no corresponding change existed over the right ventricle. No second sound heard over the left ventricle, whilst both sounds were normal over the right. At the aortic valves both sounds existed, both roughened, especially the second; the same was observed along the right side of the sternum above the third cartilage. Pulse seventy-two, moderate volume, slightly corded, bisferiens. Impulse of the heart stronger than on the fourth instant. Urine increased in quantity, less red; no precipitate from nitric acid; two alvine evacuations daily, natural, not watery.

(Digitalis discontinued, twenty-four grains taken since 29th of November.)

*December* 8.—Pulse became irregular on the night of 6th, and the double systolic sounds reappeared when excited. Double systolic movement of heart is now ninety-six per minute, while pulse at the wrist is forty-eight. Each pulsation of the heart is accompanied by a distinct thrill, more particularly marked between the third and fourth cartilages of the left side, but which is also plainly distinguishable along the right margin of the sternum, between the second and fourth ribs. Each systolic movement is accompanied by a moderate impulse, the first greater than the second; impulse greatest at point corresponding to the apex of the heart, but is also felt strongly along the left margin of the sternum, opposite the third and fourth ribs. On the line of the fourth ribs, both systolic movements are accompanied by rough or rasping sounds, especially the first; no second sound in the first systolic movement, but in the second movement, this sound exists. Over the sternum, opposite the cartilage of the third ribs, two systolic sounds are heard—the first roughened and blowing unaccompanied by any second sound, whilst the last is attended by a confused second sound, (hobbling.) Between the second and third cartilages of left side, double systolic sounds exist; in the second of which, a clear second sound is heard; the systolic sounds but slightly roughened. Between third and fourth rib, the first systolic movement is extremely rasping, the second less so; at times the second sound of the heart is observed. Marked thrill in the carotids, which beat irregularly, one pulsation being very strong, succeeded by another which is weak. Respiration more bronchial at posterior summit of left lung; tubal at the right, with sibilant rhonchus throughout. Rasping sound of heart diminished along the right margin of the spine.

On the 10th, pulse of the wrist and heart, when the patient was tranquil, coincided, ninety-six per minute. The pulsations were feeble, and became irregular upon excitement. When the heart was most calm, both cardiac sounds were heard over the aortic valves, (saw sound;) a second sound was also heard between the cartilages of the third and fourth left ribs succeeding the rasping sound which has been previously mentioned. When the heart became irregular, its pulsations increased in violence, the first sound over the sternum was then exceedingly roughened, almost rasping; the second very indistinct, but blowing; whilst the rasping sound between the third and fourth left ribs, was more intense, and the second was not heard; the sounds over the pulmonary valve remained nearly normal. A cough which had existed at times became much worse; the sputa consisted of a clear fluid slightly aerated, of yellow muco-purulent flocculi, and a third portion of small masses, of a grayish aspect, sinking to the bottom of the spitting cup.

On the 12th, pulse small, corded, requiring strong pressure to extinguish it, one hundred and eight per minute. Respiration laboured and costal, forty. Tubal respiration at posterior summit of right lung much diminished; bronchial respiration more strongly marked over the posterior upper half of the left chest, and the rough sound of heart heard more plainly along the left margin of the spine than on the right.

The ascites and anasarca having greatly increased, patient was tapped on the 13th; thirteen pints of fluid were drawn off, which proved to be nearly pure albumen. On the 14th, complained of tenderness of the abdomen, with increased heat of skin, and very small chorded pulse. (Sixty leeches applied to abdomen, followed by hop poultices. Calomel gr. ½; pulv. opii. gr. ⅙, q. b. h. Mercurial frictions to the body, and continued until slight ptyalism occurred, when there was a decided melioration of the symptoms.)

On the 19th of December it was noted, that her general health was much better; expression of countenance cheerful; disposition lively; less dyspnœa; no unpleasant dreams at night; strength increased; greater motion of the limbs; appetite good; thirst moderate; skin warm, soft, natural temperature; very slight moisture in the axilla; anasarca of lower limbs; œdema of face; abdomen considerably distended by ascites; abdomen not painful on pressure.

Impulse of heart much diminished since tapping; sounds as before, both sounds being heard over left ventricle when the patient is perfectly tranquil, but when agitated, the rasping systolic sound is only heard. Sounds normal over the right ventricle; over the aortic valves, the first sound is roughened, predominates over the second, which is short, a little blowing, and becomes indistinct upon any mental agitation. Rasping sound has ceased entirely along the right side of the sternum, but is loudly heard along the left inter-scapular space, where percussion is dull, and respiration rude, with strong resonance of the voice.

The secretion of urine, which had previously been very scanty, of a deep red colour, and turbid with red flocculent deposit, which disappeared upon the application of heat, became very copious after the exhibition for some days of copaiba in combination with digitalis and squills. The urine became of a light straw colour, free from deposit, and not yielding, as previously, a precipitate with nitric acid. The serous infiltration rapidly diminished, and in two weeks the dropsy had disappeared from the cellular tissue of the body, and from the abdomen. Convalescence during a short time seemed fully established, and all treatment was discontinued. During this mitigation of the disease, the heart beat with more regularity, both sounds being heard over the entire cardiac surface; but the moment that any cause, whether moral or physical, induced rapid pulsations of the heart, the rasping sound was again heard at the mitral valve, and at the apex; the second sound at the aortic valves became more or less masked, frequently indistinct, and the first sound at the same point much rougher than usual. Nothing anormal was discovered over the right division of the heart.

Sudden recurrence of the distressing thoracic symptoms took place, followed by rapid distention from ascites.

On the 12th of January the heart beat very feebly and tumultuously; absence of the second sound, and rasping during the systole on the left side.

Next day, whilst the patient was eating her breakfast, she fell backwards and expired.

*Autopsy, forty hours after death.*

Slight rigidity of limbs; no emaciation; serous infiltration of the face and lower extremities; abdomen distended, flat on percussion, fluctuation by palpation; thorax yields the same sound on the right side as noted during life, whilst the left chest upon being percussed is flat, except in the upper anterior portion, which is resonant.

*Chest. Right side.*—Strong adhesion of the lungs to the ribs by firm membranous attachments, especially along the spine, where the false membrane is half an inch in thickness. Upper and middle lobes much compressed and shrunken; the parenchymatous structure, which is of a mottled appearance from different degrees of sanguineous congestion, and presenting numerous capillaries filled with black blood, is friable, but not hepatized. Superior margin of the upper lobe rounded from dilated vesicles. No tubercular deposit in either of these lobes.

*Lower lobe.*—In the lower half of this lobe are several groups of unsoftened tubercles, disseminated as the air vesicles of the bronchial terminations, and apparently deposited in them; each of the larger tubercles is one twelfth of an inch in diameter, presenting a central capillary orifice. The appearance of these tubercular deposits is similar to those represented in pl. 1, fig. 1st and 3d, of Carswell's Pathological Illustrations of Tubercle. Around the larger tubercles, which are in the lower portion of the lung, the pulmonary tissue is granulated, friable, of a grayish colour, passing into the third stage of pneumonia; whilst around the smaller, and evidently more recent granulations, the lung is merely hardened and of a bright red colour. The lining membrane of the bronchial divisions communicating with the tubercles is thickened, and of a bright arterial redness.

*Left chest* contains three quarts of sanguinolent serum; no adhesion of the lungs to the ribs. Vesicles dilated on the superior margin of upper lobe; no tubercles in either lobe. The lung is much congested with bloody and spumous serosity; its parenchymatous structure is generally of a bright red, intermixed with some patches of a darker hue. The bronchi filled with frothy serosity; mucous membrane red and thickened.

*Heart.*—Pericardium distended; contains six ounces of citron-coloured fluid, in which float yellow flocculi; no adhesion of the heart to the sac. The tissue investing the heart thickened, opaque, with opaline patches on its surface. The heart is greatly enlarged by the dilatation of its right cavities; the right auricle, between the second and third ribs, extends more than two inches to the right of the sternum, whilst its summit reaches nearly to the clavicle.

*Right ventricle* is nearly double the usual size, and extends an inch beyond the right margin of the sternum, whilst the apex is an inch beyond the left nipple, corresponding with the percussion during life. The walls of the right ventricle are three lines in thickness, and it is distended by soft, semi-fluid, yellow coagula, intermixed with portions of black blood; the columnæ carneæ hypertrophied, double usual size; circumference of the auriculo-ventricular orifice, four and a half inches; tricuspid valve flexible, transparent, but does not close the orifice; regurgitation would take place through a central opening of one half of an inch. Parietes of right auricle thickened; muscular tissue measures nearly two lines. Pulmonary valves transparent, perfectly flexible, circumference three and a half inches.

*Left ventricle.*—Walls of a pale colour, six lines thick in the middle, seven and a half at the base; its cavity slightly dilated, and contains greenish-yellow gelatiniform coagula, closely adherent to the internal parietes; the endocardium is thickened, of a milky appearance; columnæ carneæ somewhat enlarged; yellow points of lymph are seen upon their surface beneath the endocardium. The mitral valve is irregularly thickened by cartilaginous deposit, especially on the free edges; the chordæ tendineæ, with one exception, are of the usual length; no regurgitation would probably take place when the heart was contracting vigorously and regularly; auriculo-ventricular orifice three and a half inches in circumference. *Aortic valves* thickened, their flexibility impaired; numerous minute granulations exist on one of the valves at its free margin; circumference of the aortic opening, two and three-quarter inches.

In the *aorta* are a few minute white patches beneath the serous coat, and with the exception of slight dotting by redness in points, the internal appearance of the vessel normal. *Pulmonary artery* normal.

*Abdomen* contains three gallons of clear yellowish serum, in which float some flocculi; a few adhesions by coagulable lymph exist between the different portions of the peritoneum; one of the bands passes from the point where puncturing was practised a few weeks since; the peritoneum near the orifice is thickened, but without anormal redness. The peritoneal coat of the liver presents numerous opaline patches of various thickness, giving to the exterior of that organ a singular marbled appearance. The *liver*, which is nine inches long, seven and a half wide, and two and a half thick, is very dense and heavy, its blood-vessels greatly engorged, its tissue hard, resisting the knife, and the brown substance is interspersed with small, circular, yellow masses, (acini?) with central depressions resembling the orifices of small vessels. *Gall-bladder* distended by viscid, yellow bile.

*Stomach.*—Mucous coat softened; strips cannot be raised by traction; numerous red points seen in the mucous coat in the cardiac extremity.

*Intestines* distended by gas, pale, and in the parts which were examined, the mucous coat was normal.

*Mesenteric glands* enlarged; some contain tubercular deposit; one of the larger glands is of the size of a walnut, containing tubercle of cheese-like colour and consistence.

*Kidneys.*—Cortical substance, in some portions, is slightly granulated.

*Spleen and pancreas* normal.

*Bladder* partially distended by urine, which is not coagulable by heat.

*Uterus* normal.

*Brain* not examined.

*Remarks.*—Many interesting facts are connected with this case. A young female of seventeen, who, with the exception of measles a year before, had enjoyed uninterrupted health, is attacked with general articular rheumatism; during the continuance of this disease she is suddenly seized with violent pain in the præcordial region, dyspnœa, and all the rational symptoms of endocarditis and pericarditis, the existence of which have been fully proven by the examination after death. Soon after the cardiac disease is manifested, effusion takes place in the cellular tissue, and is succeeded by the physical signs of enlargement of the liver and abdominal dropsy. Coincident with the appearance of ascites, the diminution in the urine was noticed, which secretion, agreeably to the analysis of an excellent chemist,* was loaded with rosacic and purpuric acids, but contained no albumen.

That the disease of the liver and kidneys was secondary to the disease of the heart, we have the best evidence; the patient, whose intelligence and memory were good, had no recollection of having had any disease previous to the measles, and her health for months before the articular rheumatism was excellent; all the secretory functions being perfectly natural. Although the change in the structure of the kidney be not great, it is of the character which marks the granulation described by Dr. Bright; and this case, in connection with those reported to this Society, goes far to show that the triple lesion of the heart, liver, and kidney, in disease of the central organ of circulation, if not constant, must, at least, be of very frequent occurrence.

The tubercular deposit, occurring only in the lower lobe of the lung, is remarkable for its rarity, and interesting from its seat in the vesicles.

But, what claims our attention as diagnosticians most strongly is, the rough bellows or rasping sound, which was heard over the left portions of the heart. This anormal murmur was very strongly marked among the first diseased cardiac symptoms; it remained constant until some mitigation took place in the violence of the disease, and subsequently, although it ceased when the patient was perfectly tranquil, yet, the slightest mental or physical cause would re-induce it. This sound was invariably heard over the mitral valve, more strongly at the apex, but never over the right division of the heart. To what cause was this sound owing? It could not have been generated in the right ventricle or its communicating valves, for over these the normal sounds were not changed; it could not have been produced at the aortic valves, for there the cardiac sounds were frequently almost natural, when the intense systolic rasping sound was heard at the upper and left lateral portions of the left ventricle, and at the apex.

It would seem, then, that this rasping sound, which occurred in the systolic movement of the heart, was produced by the regurgitation of the blood through the mitral valve into the left auricle, and that it was heard loudly at the apex, in consequence of the sound being conducted there by the columnæ carneæ. From inspection of the valve, it is evident that the left auriculo-ventricular orifice might, if the heart were contracting regularly and forcibly, be perfectly closed; but when the contractions were weak, irregular, or spasmodic, as the valve is of unequal thickness, portions of it would remain open, and through these unclosed openings the blood would be propelled during the systolic movement.

When the patient was under the influence of digitalis, the systolic action was very remarkable, a double cardiac systole existing to one pulsation at the wrist. No second sound of the heart took place after the first systolic movement, but was heard accompanying the second. A portion of blood, however, was thrown into the aorta at the first contraction, for a rough sound then existed at the aortic valves, and pulsations, though weak, were felt

* Mr. D., Apothecary to the Philadelphia Hospital.

in the carotid arteries, attended by a marked thrill. Could the formation of coagula in the ventricles of the heart produce these singular systolic actions ?

Examination of the tricuspid valve shows that it could not have formed a perfect septum between the auricle and ventricle during the systole, but that regurgitation must have taken place; yet, no anormal sound existed over the right division of the heart. I have frequently observed that *mere deficiency* in the valvular closure of the right osteum venosum, did not induce any morbid sound, and have regarded it as collateral evidence of the correctness of the opinion that the tricuspid is a valve of safety, allowing regurgitation during periods of momentary excitement, and thus preventing too great an afflux of blood upon the lung, which would otherwise occur. When, however, any roughness or thickening exist in the valve, then, if the current of blood passing backwards be driven with force, anormal sounds take place.

